# Mitigating Identity-Related Anxiety through Humor and Immersive Storytelling with 360-Degree Video in Virtual Reality: A Study on Microaggressions’ Mental Health Effects

**DOI:** 10.3390/ijerph21060713

**Published:** 2024-05-31

**Authors:** Changmin Yan, Alan Eno, Adam Wagler

**Affiliations:** College of Journalism and Mass Communications, University of Nebraska-Lincoln, Lincoln, NE 68588, USA; alaneno@unl.edu (A.E.); adamwagler@unl.edu (A.W.)

**Keywords:** microaggressions, virtual reality, humor therapy, immersive storytelling, identity-related anxiety, narrative persuasion, character identification, psychological presence, diversity, inclusion

## Abstract

**Background**: Microaggressions are subtle slights that can cause significant psychological distress among marginalized groups. Few studies have explored interventions that might mitigate these effects. **Objective**: This study aimed to investigate if and how humor-infused immersive storytelling via virtual reality (VR) could reduce identity-related psychological distress caused by microaggressions. **Methods**: Using a community-based participatory research approach, we developed a 7-min 360-degree VR film depicting scenarios of microaggressions across various identities. Forty-six college students participated in a controlled study where they were exposed to this immersive VR experience. We measured identity-related psychological anxiety, character identification, perceived humor, and perceived psychological presence. **Results**: The findings demonstrated a significant anxiety reduction following the VR intervention, supporting the efficacy of humor-infused storytelling in alleviating the impact of microaggressions. Character identification significantly predicted anxiety reduction, while perceived humor and psychological presence did not directly influence anxiety reduction but indirectly contributed through enhanced character identification. **Conclusions**: Humor-infused immersive storytelling, facilitated by VR, effectively reduces identity-related psychological distress primarily through character identification. The structural equation modeling results emphasize the importance of integrating humor and psychological presence to enhance character connection, advocating for a balanced approach that combines traditional narrative elements with technological innovations in health interventions aimed at combating the adverse psychological effects of microaggressions.

## 1. Introduction

Microaggressions, though usually understated, can bring about profound discomfort for marginalized communities. Such conduct, whether knowingly perpetrated or not, emits harmful, disparaging, or negative racial nuances and criticisms that may potentially lead to detrimental or distressing psychological effects on the targeted individual or group [1]. The ramifications of microaggressions on mental health are tangible and enduring among the victimized group. As expressed by a group of Lesbian, Gay, and Bisexual (LGB) youth, subtle LGB affronts felt like enduring “death from a thousand cuts” [2]. Racial microaggressions erode self-esteem and impede academic and workplace performance among Asian Americans [3,4] and are associated with psychological distress and escalated risks of heart disease among African Americans [5]. However, addressing microaggressions can evoke discomfort. Could laughter possibly alleviate this? By co-mingling humor-infused storytelling and immersive technologies, this study aims to address two primary questions: (1) Can exposure to humor-infused immersive storytelling about microaggressions alleviate identity-associated psychological distress? (2) What are some of the contributing factors for the hypothesized reduction of identity-based psychological distress?

This paper is structured to facilitate a clear understanding of the study and its findings. Following this introduction, “Related Work and Hypotheses Development” in Section 2 provides a detailed review of the existing literature and delineates the hypotheses tested in this study. Section 3, “Materials and Methods”, describes the experimental design, participant selection, data collection methods, and analytical techniques employed. Section 4, “Results”, presents the findings of the study. Section 5, “Discussion”, interprets the results. Section 6 discusses practical implications. Section 7 focuses on limitations and suggests directions for future research. Finally, Section 8, “Conclusions”, summarizes the key findings and the contributions.

## 2. Related Work and Hypotheses Development

### 2.1. Effects of Microaggressions on Marginalized Groups

Marginalized groups are “groups and communities that experience discrimination and exclusion (social, political and economic) because of unequal power relationships across economic, political, social and cultural dimensions” [6]. Marginalization creates barriers for certain groups from accessing resources, opportunities, and rights that are readily available to the rest of society based on their race, ethnicity, socioeconomic status, gender, sexual orientation, disability, and social, political, or cultural affiliations [7]. Marginalization can occur along multiple dimensions at once, for instance, in regard to sexual orientation and ethnicity [8].

Numerous studies explore the impact of microaggressions on marginalized communities. Microaggressions typically manifest as “brief and commonplace daily verbal, behavioral, or environmental indignities, whether intentional or unintentional”, and can inflict considerable harm upon individuals of marginalized groups [3]. Individuals may experience distress when their self-concept is called into question, an effect notably prevalent in marginalized identities [9]. Researchers investigating this association between microaggressions and identity-related distress have found that continuous exposure to microaggressions can lead to significant psychological distress, such as anxiety, depression, and low self-esteem, particularly among ethnic minority groups [2]. The subtle nature of these affronts makes them challenging to challenge and resist, thereby inciting a sense of helplessness and frustration. Research reports on microaggressions, and the mental health of college students of color reveal that microaggressions increased distress and created an unfriendly learning atmosphere, leading to lower retention rates in their chosen fields of study [10].

Researchers have also noted the intersectionality of microaggressions [8,11]. In a study to understand how microaggressions might intersect with other forms of oppression, such as sexism, homophobia, and classism, individuals at the crossroads of multiple marginalized identities experienced distinct and more intense forms of microaggressions, leading to escalated levels of identity-related distress [11].

### 2.2. Humor as a Coping Mechanism

While subtle gestures or remarks marginalizing certain social groups are known to cause distress and anxiety, there is increasing evidence to suggest that humor can act as a mitigating factor. Some researchers proposed the concept of humor serving as a stress buffer, implying that individuals equipped with a robust sense of humor might be less prone to distress during stressful situations [12]. Other scholars explored this further, examining humor’s role in tempering anxiety derived from racial microaggressions [13]. They found that individuals capable of reinterpreting microaggressions in a humorous light reported reduced anxiety and distress. Parallel to this, humor has been found to be a protective shield against the detrimental impacts of gender-related microaggressions [14]. Women using humor as a strategy to cope with sexist microaggressions experienced reduced stress and anxiety levels [14]. Humor, particularly forms of humor that involve absurdity or reframing situations can be used as a tool to resist and challenge the harmful narratives perpetuated by microaggressions [15]. Such findings highlighted the empowering potential of humor in the face of marginalization.

### 2.3. Immersive Storytelling as an Intervention Tool

Research on immersive storytelling intersects narrative persuasion theory, digital technology, and user experience studies. The promise of immersive storytelling is to engage audiences more deeply through multi-sensory, interactive experiences that might blur the line between reality and fiction.

Immersion involves the audience’s cognitive and emotional engagement in the narrative and their willingness to momentarily suspend disbelief to accept the narrative world as their temporary reality [16]. Using digital technologies, “multiform stories” could be reshaped and retold in response to user input [17]. This concept has been realized in a variety of immersive media forms, from video games to virtual and augmented reality. Virtual reality (VR) has been used to create immersive narratives as mental health treatment [18]. VR can “transport” users into different environments and significantly increase the level of immersion and emotional engagement, thereby transforming the narrative experience and delivering psychological therapies [18].

Several scholars have researched the psychological impact of immersive storytelling on audience perceptions. Some found that immersive environments could lead to a sense of “presence”, in which users feel as if they are personally “inside” the narrative world [19]. Others explored the psychological impact of immersive storytelling, finding that it can lead to higher levels of empathy and understanding by allowing users to “experience” perspectives other than their own [20].

Given the mitigating potential of humor and the transformative user experience of immersive storytelling, it is reasonable to hypothesize the positive effect of co-mingling humor-based storytelling and immersive technologies.

**H1.**  *Exposure to humor-based immersive storytelling about microaggressions will reduce identity-related psychological distress.*

**H2.**  *Perceived humor will positively predict a reduction in identity-related psychological distress.*

### 2.4. Underlying Mechanisms of Reducing Identity-Related Psychological Distress

#### 2.4.1. Character Identification in Humor-Based Storytelling

If humor could indeed help with identity-related anxiety mitigation, one of the potential mechanisms might be character identification. The stronger the identification with a character, the more likely the audience is to perceive humor consistent with the character’s experiences [21]. Even in immersive environments, humor could facilitate identification with virtual characters, subsequently enhancing the immersive experience [22]. Identifying characters that employ humor to cope with stressful situations could enhance the viewers’ own resilience and provide a way of coping with adversity [12]. Therefore, it is plausible to anticipate a contributing role of character identification in the hypothesized distress-reduction by exposure to humor-based immersive storytelling about microaggressions among marginalized groups.

**H3.**  *Character identification will positively predict a reduction in identity-related psychological distress.*

#### 2.4.2. Immersive Technologies

Immersive technologies, such as VR and 360-degree videos, offer new opportunities for understanding psychological presence, i.e., the sense of “being there” in a situation or environment and how it impacts a user’s cognitive and emotional responses [19]. Psychological presence in immersive technologies can enhance learning outcomes [23,24] and help patients cope with fears and phobias in a safe, controlled setting [25]. Concerning its impact on identity-related anxiety, mindfulness, which is closely related to psychological presence, can lead to greater self-insight and self-acceptance, which could positively impact one’s identity and self-concept [26]. Mindfulness and acceptance practices could reduce identity-related anxiety, particularly among individuals with marginalized identities [27]. Similarly, mindfulness-based cognitive therapy could alleviate symptoms of identity-related anxiety by enhancing psychological presence and promoting non-judgmental acceptance of self-identity [28]. As such, the following contributing role of psychological presence can be proposed.

**H4.**  *Psychological presence will positively predict a reduction in identity-related psychological distress.*

## 3. Materials and Methods

### 3.1. Stimuli Development

#### 3.1.1. Microaggression Themes

Taking a Community-Based Participatory Research (CBPR) approach [29], 46 college students from diversity and inclusion classes were recruited to share their stories of microaggression in the needs assessment stage. They were recruited to participate in this study through purposive sampling, targeting individuals from diverse backgrounds to ensure a wide range of experiences concerning microaggressions. The participants were from various academic disciplines and represented multiple identities in terms of gender, race, language/nationality, disability, and cultural background. Informed consent was obtained from all participants, and the study protocols were approved by the institutional review board (IRB) of the university.

During the planning stage, an expert panel comprised of faculty, staff, and students from the College of Journalism and Mass Communications and the university’s Office of Diversity and Inclusion was assembled to conduct a thematic analysis of the students’ experiences of microaggression. The same expert panel scripted and produced a 7-min 360-degree short film to cover five themes of microaggressions, including gender, language/nationality, race, disability, and culture.

#### 3.1.2. Data Collection

Participants were engaged in a series of focus group discussions designed to elicit their personal experiences with microaggressions in a college setting. Each focus group was facilitated by a trained moderator using a semi-structured interview guide, which included prompts and questions aimed at uncovering detailed narratives about specific incidents of microaggressions related to the five identified themes: gender, language/nationality, race, disability, and culture.

The qualitative data collected from these discussions were transcribed verbatim and subjected to thematic analysis. An expert panel consisting of researchers proficient in qualitative methods and knowledgeable about microaggressions reviewed the transcriptions independently. The panel then identified and categorized prevalent themes. Discrepancies in thematic interpretation were resolved through consensus. A script was subsequently written to capture the five identified themes in a short film (see Supplementary File S1 for the film script).

#### 3.1.3. Development of the VR Film

Based on the film script, a 7-min 360-degree VR film was produced to encapsulate the five prevalent themes of microaggressions. The film was scripted collaboratively by a team comprising researchers, participants, and a VR production team. The VR scenario was designed to immerse viewers in real-life situations of microaggressions, as described by college student actors. This immersive experience aimed to foster empathy and understanding among broader college audiences regarding the subtle yet impactful nature of microaggressions in academic environments. This comprehensive approach ensured that the study was grounded in the lived experiences of the participants while utilizing innovative technology to enhance awareness and education on microaggressions in college settings.

The film recording space was an approximately 500 square-foot editing bay in the basement of a rented space that the University’s student advertising agency used for their multimedia production crew, and it gave us enough room to spread the actors apart since this was filmed during the first year of the COVID-19 pandemic. The location also allowed for access control, so there were no unexpected interruptions. Six actors were spaced in a ring of seven chairs six feet apart and were masked between takes. To boost the viewer’s immersive experience, the camera was placed at the spot of the seventh chair in the ring and adjusted to the average height of the actors’ eyes. The script was written for a six-to-eight-minute run-time and was filmed as a single take.

To capture the video footage, we used a tripod-mounted Insta360 Pro, which has four spatial audio microphones and six cameras that record 8K (7680 px × 4320 px) at 30 fps to a 1 tb USB-C SSD. The footage was then transferred to the Insta360 Stitcher application to unify all six camera frames into a single 8K video clip. Six Sennheiser EW 122P wireless Lavalier mic kits were connected to a Zoom H6N audio recorder to capture each individual actor on an independent audio track for additional spatial audio options. Since the nature of 360 videos discourages time and framing edits, the entire story was shot as a single take, and six total takes were recorded.

The story was edited in Adobe Premiere Pro as an 8K 29.97 fps Square pixel (1.0) progressive scan sequence with seven audio tracks (6 actor voice tracks plus a nat soundtrack) mixed for realistic sound from the point of view of a group participant. The pilot draft was not edited to make full use of the spatial audio. However, the individual voice tracks were only mixed for level and sound balance with the nat soundtrack. The finished edit ran just under seven minutes (6:56:00) and was rendered as a 2:1 aspect ratio 8K (7680 px × 3840 px) 29.97 fps (square pixel, progressive scan) MP4 file with a high VBR two-pass H.264 compression (12.0 Mbit/s average) and a single stereo AAC 48 khz audio track to maximize video and sound quality when in the VR headset.

### 3.2. Main Study on Effects of the VR Film

#### 3.2.1. Participant Recruitment

Over a three-week period, 176 students enrolled in diversity and inclusion-related courses at the university were selected to view the 7-min 360-degree VR film (41% male, 59% female; mean age = 21.71, SD = 2.52; 87% White, 5% American Hispanics, 4% African American, and 4% Asian American). Recruitment was conducted through course instructors who provided extra credit as an incentive for participation. All participating students provided informed consent, with the study’s objectives and procedures clearly explained prior to their involvement. All subjects gave their informed consent for inclusion before they participated in the study. The study was conducted in accordance with the Declaration of Helsinki, and the protocol was approved by the Ethics Committee of University of Nebraska-Lincoln (IRB project # 20220522004EX).

#### 3.2.2. Measures and Data Collection

The assessment of the impact of the VR film on the participants involved a pre-viewing baseline assessment (week 1) and a post-viewing evaluation (week 3) during a period of three weeks. The study spanned three weeks, beginning with a pre-film survey to establish baseline measures of identity-related anxiety and demographics. After a three-week interval to minimize any initial survey effects, participants watched a 7-min 360° VR film depicting microaggressions, followed immediately by a post-film survey assessing post-film identity-related anxiety, perceived humor, character identification, and psychological presence. This design allowed us to isolate the VR intervention’s effects within a controlled timeframe, ensuring the clarity and validity of the study’s findings. The following measures were administered.

#### 3.2.3. Identity-Related Anxiety and Perceived Humor

A 3-item psychic (personal identity) Existential Annihilation Anxiety (EAA) scale [30] was used to assess feelings of anxiety related to one’s identity, with students answering these questions before and after viewing the film to measure any changes attributable to the experience. The EAA scale is comprised of three 6-point items (1 = not at all to 6 = a great deal), including “(1) Because of what happened to me, I sometimes worry that I just lose my sense of self; (2) sometimes I feel the threat of extermination of my group because of discrimination; (3) I feel threatened by extreme inequalities in this society”. The Cronbach’s α is 0.89 for pre-film identity-related anxiety and 0.81 for post-film identity-related anxiety. The three items were averaged to create the EAA scale. An identity-related anxiety change score was calculated for each participant.

Perceived humor was measured by an original 2-item scale with two 7-point items (1 = not at all to 7 = a great deal), including “(1) I found the 360-degree VR film humorous; (2) To me, the 360-degree VR film was funny”. The Cronbach’s α is 0.91 for perceived humor. The two items were averaged to create the perceived humor scale.

#### 3.2.4. Character Identification

The 14-item Identification with Characters measure [31] on a 5-point Likert scale (1 = not at all to 5 = very much) was used to assess the extent to which students felt they could relate to or see themselves in the characters depicted in the VR scenarios. High scores on this scale suggest a stronger personal connection with the content and characters in the VR film. The Cronbach’s α is 0.79 for post-film perceived psychological presence. The fourteen items were averaged to create the character identification scale.

#### 3.2.5. Perceived Psychological Presence

Post-viewing, students were asked to complete a 5-item perceived psychological presence scale measuring their sense of “being there” within the VR environment, an indicator of the VR experience’s immersive quality. The five 7-point items (1 = strongly disagree to 7 = strongly agree) were adopted from the Spatial Experience Presence Scale [32] and the Presence Questionnaire [33]. They are as follows: (1) To what extent did you feel like you were really in the meeting room?; (2) to what extent did you feel surrounded by the environment (e.g., chairs, tables, other students)?; (3) I felt like I really stayed in the meeting room; (4) the video/experience felt like what would happen in the real world. (5) I felt like I could reach out and touch the objects in the video. The Cronbach’s α is 0.81 for post-film perceived psychological presence. The five items were averaged to create the perceived psychological presence scale.

### 3.3. Data Analysis

Data collected from the pre- and post-viewing assessments were analyzed using paired *t*-tests to identify statistically significant changes in identity-related anxiety (H1). Additionally, regression analysis and structural equation models were employed to test if perceived humor (H2), character identification (H3), or perceived psychological presence (H4) can predict changes in identity-related anxiety. Descriptive statistics of the measured variables are summarized in Table 1. This detailed methodological approach enabled a comprehensive evaluation of the VR film’s effectiveness in reducing identity-related anxiety among college students, providing insights into the specific aspects of the VR experience that most significantly impacted viewers.

## 4. Results

Descriptive statistics of the measured variables are summarized in Table 1. Results showed that data were normally distributed as skewness and kurtosis of each measured variable individually were within ±1. Critical ratio (Z value) of the skewness and kurtosis of each measured variable were within ±1.96, also evident to normally distributed data.

**H1.**  *Exposure to humor-based immersive storytelling about microaggressions will reduce identity-related psychological distress.*

A paired *t*-test was run to test the change in identity-related anxiety after exposure to the VR film, and the results (t (175) = 9.33, *p* < 0.001) indicate that self-reported identity-related anxiety was reduced (baseline mean = 3.70, SD = 0.94; post-film mean = 3.05, SD = 1.15). In sum, the results support H1 in that participants reported a significant reduction in identity-related anxiety after interacting with the VR film.

**H2, H3, and H4.**  *Perceived humor (H2), character identification (H3) or psychological presence (H4) will positively predict reduction of identity-related psychological distress.*

Multiple regression was employed to examine perceived humor, character identification, and perceived psychological presence as predictors of reduction of identity-related anxiety. Table 2 reports the statistics associated with this analysis and shows that together, these three variables accounted for a significant portion of the variance in the reduction of identity-related anxiety. Character identification significantly predicted a reduction in identity-related anxiety at α = 0.05, supporting H3. However, perceived humor and perceived psychological presence were not significant predictors of identity-related anxiety at α = 0.05, failing to support direct effects for H2 and H4.

To further explore the potential indirect roles of perceived humor and perceived psychological presence in reducing identity-related anxiety via character identification, a structural equation model (SEM) was estimated in R to examine the relationships among perceived humor, perceived psychological presence, character identification, and identity-related anxiety reduction. The model included direct effects of presence, humor, and identification on anxiety reduction, as well as indirect effects of presence and humor on anxiety reduction through character identification. Presence and humor were allowed to correlate. The model was estimated using the Maximum Likelihood (ML) method with 176 observations. The fit indices for the SEM indicated an adequate model fit: Chi-Square Test: Chi-Square = 5.41, *p* = 0.07; Comparative Fit Index (CFI): 0.99; Tucker-Lewis Index (TLI): 0.96; Root Mean Square Error of Approximation (RMSEA): 0.09; Standardized Root Mean Square Residual (SRMR): 0.04.

Character identification had a significant positive direct effect on anxiety reduction (β = 0.56, *p* < 0.001), suggesting that higher levels of character identification are associated with greater reductions in identity-related anxiety. Both perceived humor (β = −0.17, *p* = 0.64) and perceived psychological presence (β = −0.03, *p* = 0.71) showed a non-significant direct effect (β = −0.03, *p* = 0.71) on anxiety reduction.

The model revealed significant indirect effects of perceived humor (Z = 2.43, *p* < 0.05) and perceived psychological presence (Z = 2.12, *p* < 0.05) on identity-related anxiety reduction through character identification. These effects indicate that both perceived humor and perceived psychological presence positively impact anxiety reduction through the mediation of enhanced character identification. The covariance between presence and humor was significant (Z = 3.29, *p* < 0.001), indicating a positive relationship between these constructs within the dataset.

**Figure 1:**
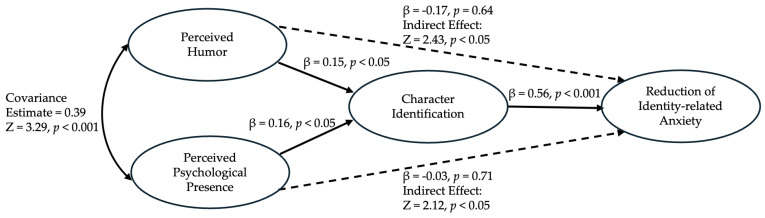
A structural equation model estimating the relationships among perceived humor, perceived psychological presence, character identification, and identity-related anxiety reduction.

These findings, detailed in Figure 1, illustrate the indirect pathway through which perceived humor and perceived psychological presence contribute to reducing identity-related anxiety, supporting indirect effects for H2 and H4. The relationships underscore an indirect effect rather than a direct influence of perceived humor and perceived psychological presence on anxiety reduction.

## 5. Discussion

### Interpretation of Findings

The results of this study indicate that humor-infused immersive storytelling can significantly reduce identity-related psychological distress among participants exposed to microaggressions. This finding supports our first hypothesis (H1) and aligns with prior research suggesting that humor can serve as a stress buffer, fostering long-term psychological resilience [12,13,14].

Significantly, the study supports the hypothesis (H3) that character identification plays a crucial role in mitigating identity-related distress. Participants who identified more strongly with characters experiencing similar challenges reported greater reductions in psychological distress. This suggests that character identification may tap into deeper emotional connections, enhancing the therapeutic impact of the narratives [21,22].

Consistent with past research [21], which highlighted how affective dispositions toward public figures can influence emotional responses to news about these individuals, our data revealed that character identification significantly predicts reductions in identity-related anxiety. Just as previous studies [21] documented that emotional engagement with characters can drive emotional responses, our study demonstrates that strong identification with characters in immersive VR storytelling can lead to reduced psychological distress. This emotional engagement is crucial for fostering empathy and allowing participants to connect deeply with the characters’ experiences.

Moreover, past research [22] demonstrated significant differences in perceived intimacy and co-presence between text-based communication and more immersive modes, such as avatars. The immersive characteristics of avatar-based communication were cited as the driving force behind the enhanced relationship between perceived co-presence or closeness with the interactive communication partner and perceived intimacy or interpersonal trust. Although our research focuses on VR storytelling rather than avatars, the underlying principle is similar: immersive experiences, whether through avatars or VR characters, enhance social presence and emotional engagement. Our findings that perceived psychological presence and character identification contribute to anxiety reduction parallel past research [22], which noted higher levels of co-presence and emotional connection in more immersive communication modes.

Echoing insights from the abovementioned past studies [21,22], our findings underscore the importance of character identification in the context of immersive VR storytelling. These connections highlight the broader applicability of character engagement across various media forms and reinforce the therapeutic potential of using immersive narratives to address psychological distress.

In addition, our results support the hypothesis (H2) that humor, when integrated into immersive storytelling through VR, can significantly mitigate such distress, primarily through mechanisms of character identification. Importantly, this intervention leverages both the immersive capabilities of VR and the psychological buffer provided by humor. Similarly, our examination of the hypothesis (H4) concerning the relationship between perceived psychological presence and reductions in identity-related anxiety reveals a more nuanced interaction between them. Perceived psychological presence indirectly influenced reductions in identity-related anxiety. The SEM estimates revealed indirect effects through character identification, suggesting that both perceived humor and psychological presence can facilitate deeper narrative engagement, which, in turn, leads to reduced anxiety. These nuanced roles of perceived humor and psychological presence support its contribution to the storytelling experience by enhancing engagement and emotional connection rather than directly influencing mental health outcomes. These findings challenge prior assertions that the immersive qualities of VR have straightforward psychological benefits [18,19,23], suggesting instead that the impact of immersive technologies may be more complex and primarily serve to enrich the narrative experience.

Some readers may raise questions regarding the specific contributions of humor, as opposed to the storytelling or VR components individually. To address this, regression and SEM analyses were conducted to explore hypothesis 2, which focuses on both the direct and indirect impacts of humor. The analyses revealed that humor does not have a significant direct effect on reducing psychological distress, suggesting that humor alone does not independently influence outcomes outside of the VR environment. However, the significant indirect effect of humor, facilitated through enhanced character identification within the immersive VR setting, indicates that humor’s beneficial effects are likely realized in synergy with the immersive qualities of the VR environment. This distinction underscores the integrated nature of the components and highlights the enhanced therapeutic potential of combining humor with VR storytelling.

## 6. Implications for Practice

These findings have important implications for diversity training programs and identity-related mental health interventions. They suggest that integrating humor and immersive storytelling can address sensitive issues like microaggressions effectively, reducing associated distress. However, the key to these interventions appears to be in good old-fashioned storytelling and satires and the ability of narratives to foster deep emotional connections through character identification [12,15], rather than the technological novelties of VR alone.

The SEM results further illuminate the unique contributions of perceived humor and psychological presence in enhancing engagement through character identification. Such insights suggest that while the technological facets of VR contribute to the narrative experience, their primary role is to facilitate deeper connections with the content rather than directly alleviating psychological distress. Practitioners should focus on developing VR experiences that not only captivate audiences but also foster strong identification with characters experiencing microaggressions. This can be achieved by carefully crafting character arcs and narratives that resonate deeply with participants’ own experiences or by portraying relatable challenges that elicit empathy and understanding. Such strategies can enhance the emotional impact of the stories and promote psychological resilience among viewers.

Moreover, these findings indicate that while the “wow” factor of VR is substantial, the real value in using such technology in educational and therapeutic contexts lies in its ability to deepen narrative engagement. Therefore, training programs and mental health interventions employing VR should prioritize narrative development and character relatability to maximize the therapeutic benefits of these interventions.

Humor can serve as a useful tool to facilitate engagement and reduce psychological distress by creating a more relatable and enjoyable experience for participants. Practitioners should consider incorporating humor into VR narratives, as it can significantly enhance character identification and emotional connection, leading to greater therapeutic outcomes. When designing VR interventions, it is crucial to integrate humor in a way that aligns with the overall narrative and character arcs. This integration can help make serious topics, such as microaggressions, more approachable and less intimidating, thereby encouraging participants to engage more deeply with the content. Humor should be used thoughtfully to complement the story and not detract from the gravity of the issues being addressed. Furthermore, humor has the potential to break down barriers and build rapport between participants and the characters, fostering a sense of empathy and understanding. This can be particularly beneficial in educational and therapeutic settings where the goal is to promote psychological resilience and emotional well-being. By leveraging humor alongside technological advancements in VR, practitioners can create more effective and impactful interventions. The combination of immersive storytelling and humor can provide a holistic approach to addressing identity-related psychological distress, making the experience both meaningful and enjoyable for participants.

In practice, researchers and health professionals are encouraged to collaborate with storytellers and technologists to create immersive narratives that are not only technologically advanced but are also emotionally compelling and pedagogically sound. This collaborative approach can ensure that the use of VR in addressing microaggressions and other sensitive topics effectively supports learning and personal growth.

## 7. Limitations and Future Research

This study’s limitations include its reliance on a college student sample and self-reported measures of distress, which may introduce bias and affect generalizability. Additionally, the indirect role of psychological presence found through mediation analysis suggests that future research should further explore the mechanisms through which VR enhances narrative engagement and reduces distress. It is critical to validate these indirect effects with a more diverse demographic and use objective stress indicators to confirm the findings.

Further investigation should also focus on different narrative elements and emotional responses that could either enhance or mitigate the impacts of microaggressions. The long-term effects of repeated exposure to such VR storytelling interventions merit exploration as well to assess the sustainability and broader applicability of these techniques in diverse settings.

This study was designed to integrate humor within the VR storytelling experience without distinct control conditions for each component. This design choice focuses on examining the combined effect of these elements rather than isolating their individual contributions. While this approach limits our capacity to definitively parse out the specific impact of humor, storytelling, or VR immersion alone, the regression and SEM results do provide valuable insights into the effectiveness of the integrated intervention. Future studies could build on these promising findings by incorporating separate control conditions to further clarify the individual contributions of each component.

Findings from this research contribute significantly to our understanding of the therapeutic potential of humor-infused immersive VR storytelling in reducing identity-related psychological distress. However, it also recognizes the limitations in the scope of interventions tested. While our findings highlight the unique benefits of a VR intervention, such as enhanced emotional engagement and narrative immersion, we acknowledge that simpler, more accessible methods, such as passive VR exploration or traditional social interactions, were not comparatively evaluated. It is worth noting that, once developed, VR-based interventions can be scaled up for widespread, repeated use at minimal additional cost, making them more economical in the long term compared to interpersonal interactions that require ongoing time and labor investment. Leveraging the initial VR investment, these interventions can provide a cost-effective solution for addressing psychological distress on a larger scale. Nevertheless, future research should include a broader array of control conditions, comparing these less resource-intensive methods directly against our VR storytelling approach. This expanded analysis will allow us to assess the effectiveness of various interventions, ensuring that our recommendations are both scientifically robust and practically applicable across different settings, thus striking a balance between innovation and accessibility.

A potential limitation of our study is the selection bias inherent within our sample, which consisted of students enrolled in diversity and inclusion-related courses. This group’s pre-existing interest in and familiarity with the topics of diversity and inclusion might have influenced their responsiveness to the intervention. Such students are likely to have a heightened awareness and potentially a greater sensitivity to issues related to microaggressions, which could affect their perception and evaluation of the humor-infused immersive storytelling experience presented in the study. While this provided a relevant and engaged sample for exploring the impact of our intervention on identity-related psychological distress, it also limits the generalizability of our findings to a broader population. To mitigate this concern and enhance the external validity of our research, future studies should seek to replicate these findings across diverse educational settings and among student populations without a direct academic focus on diversity and inclusion. This would help determine whether the observed therapeutic benefits of our intervention are consistent across different demographic and psychographic profiles, thereby providing a more comprehensive understanding of its efficacy and applicability.

We recognize that participants’ prior experience with VR could influence their sense of the novelty effect of the VR technology and potentially play a role in how they engage with and perceive the immersive story. Although we did not measure this in the current study, it presents an opportunity for future research to explore. Future studies should consider assessing participants’ previous VR exposure to better understand its potential moderating role. Including a diverse sample with varying levels of VR experience could also help generalize the findings more broadly.

## 8. Conclusions

The findings of this study highlight the complex interplay between technology and narrative in mitigating identity-related psychological distress. While VR technology significantly enhances psychological presence, the mediation results reveal that the actual mitigating effect on distress stems from the depth of character identification. These results emphasize the enduring power of narrative to foster empathy and psychological resilience, suggesting that technological advancements should complement rather than supplant the fundamental elements of effective storytelling and satirizing microaggressions.

In conclusion, as we continue to leverage emerging technologies like VR for educational and therapeutic purposes, it is crucial to maintain a focus on the quality of storytelling, the emotional responses, the psychological presence, and the narrative engagement it fosters. This study not only supports the use of VR in addressing microaggressions but also points to the importance of integrating sound narrative practices to fully realize the potential of these technologies.

## Figures and Tables

**Table 1:** Descriptive Statistics of Measured Variables.

Variables	Mean	Median	Variance	Standard Deviation	Range	Skewness	Kurtosis
Baseline Identity-related Anxiety	3.70	3.67	0.89	0.94	3.67	0.20, SE = 0.18, Z = 1.11	−0.60, SE = 0.36, Z = −1.67
Post-film Identity-related Anxiety	3.05	3.00	1.33	1.15	4.50	0.01, SE = 0.18, Z = 0.06	−0.48, SE = 0.36, Z = −1.33
Perceived Humor	5.02	5.00	1.00	1.42	4.50	−0.25, SE = 0.18, Z = −1.39	0.52, SE = 0.36, Z = 1.44
Character Identification	4.41	4.40	1.12	1.06	3.80	−0.33, SE = 0.18, Z = −1.83	−0.70, SE = 0.36, Z = −1.94
Perceived Psychological Presence	3.83	3.80	1.09	1.04	3.80	0.15, SE = 0.18, Z = 0.83	−0.67, SE = 0.36, Z = −1.86

**Table 2:** Predictors of Reduction of Identity-related Anxiety.

Variables	β	t	Sig.
Perceived Humor	−0.17	0.47	0.64
Character Identification	0.56	8.29	<0.001
Perceived Psychological Presence	−0.03	−0.37	0.71

F (3, 172) = 23.37, Adjusted R-Sq = 0.28, *p* < 0.001.

## Data Availability

The datasets presented in this article are not readily available because we do not have IRB approval to share the data. Requests to access the datasets should be directed to cyan3@unl.edu.

## Supplementary Materials

The following supporting information can be downloaded at: https://www.mdpi.com/article/10.3390/ijerph21060713/s1, File S1: VR Film Script.
