# Supplementary material for: Mitigating Identity-Related Anxiety through Humor and Immersive Storytelling with 360-Degree Video in Virtual Reality: A Study on Microaggressions’ Mental Health Effects
Source: Int J Environ Res Public Health. 2024 May 31;21(6):713. doi: 10.3390/ijerph21060713 (PMC11203420; doi:10.3390/ijerph21060713)
Supplement: Supplementary file 1 [file ijerph-21-00713-s001.zip › ijerph-3010369-supplementary.pdf]

VR Film Script

Characters

Actor 1: Qizhen (Asian-American student)
Actor 2: Carlos (Hispanic-American student)
Actor 3: Jessie (International student)
Actor 4: Eric (Trans-Female student)
Actor 5: Chad (DudeBro)
Actor 6: Moon(Hearing disability)

YOU: the viewer

INT. DOWNTOWN LINCOLN - AFTERNOON

Some office space owned by the University somewhere in downtown. The room doesn’t serve a purpose yet, so it’s a nice quiet place for someone to get work done.

You, the viewer, are a member of a small group of students working together on a group project. Every member of your group comes from different backgrounds, so there is sure to be something new to learn from one another.

SCENE: Introduction

ERIC
Alright. Is everyone here yet or are we still waiting on someone?

QIZHEN
Carlos just messaged the group chat; says he’s just parked.

JESSIE
Oh good. We can just wait for him then.

CHAD
So does anyone have any idea about what we’re supposed to do for this paper?

QIZHEN
I’m not quite sure. I haven’t had a chance to look at the syllabus yet. We can talk more about when Carlos gets here.

CARLOS
Hey guys!

EVERYONE
Hey!

CARLOS
Sorry I’m late; my class ran a little longer than usual. I didn’t miss anything did I?

QIZHEN
Oh no you’re fine. We were waiting on you to get here before we got started anyway. Have a seat.

CARLOS
Oh, well you guys didn’t have to wait for me…

ERIC
It wasn't a problem; You weren't that late anyway.

CARLOS
Sounds good.

ERIC
Ok, so I know this might seem dumb to some of you, but I'd like to go around the table and introduce ourselves. I get that ice breakers aren't everyone's thing, but it wouldn't be a bad idea to get to know one another since we'll be working together for awhile. Is that alright with everyone?

EVERYONE
Sounds good to me.

QIZHEN
I can start if you want.

JESSIE
Sure, go ahead.

SCENE 1: Asian Culture

QIZHEN
Ok. Hey guys. My name is Qizhen.

JESSIE
"Chee-gen"?

QIZHEN
No Qizhen.

JESSIE
What?

QIZHEN
Qizhen. Like Key Hen.

JESSIE
"Chee-gen."

QIZHEN
Yeah that's close enough.

CHAD
Is it ok if we just call you Hen? That's easier to say.

QIZHEN
You can just call me Key, if that’s better.

ERIC
Interesting name. It’s not a very common around here. Where are you from?

QIZHEN
I was born here in Lincoln, but my mom’s family is from the Philippines.

CARLOS
Well, your English is really good.

QIZHEN
Thanks. Yours is too.

CHAD
I have an uncle who went to the Philippines once. Came back with a cute little wife.

EVERYONE stares uncomfortably at CHAD.

CHAD
What? What did I say?

JESSIE
By the way Key, don’t take this the wrong way, but can I ask you something?

QIZHEN
Sure, what’s your question?

JESSIE
Is it true Filipinos eat dog meat?

EVERYONE reacts with simultaneous responses.

SCENE 2: Hispanic Culture

ERIC
Let’s just move on. Who wants to go next?

CARLOS
I’ll do it.

ERIC
Thank you.

CARLOS
I’m Carlos. I’m from California, but I went to high school in Colorado.

QIZHEN
How come?

CARLOS
I don’t know. Something about my dad’s in the military, so we had to move around a lot. Mom wanted to move back so she could be close again with the family, so they did about a year-and-a- half ago.

CHAD
How did you sleep with all the ambulances and police sirens?

CARLOS
What do you mean?

JESSIE
I’ve just heard L.A. is real dangerous

CARLOS
I mean, there are some dangerous areas, but that’s any city.

CHAD
Just saying it seems like a hard place to raise a big family.

CARLOS
I’m an only child.

CHAD
Oh… Sorry, I just assumed that…

CARLOS
What? You just assumed because I’m hispanic, you think we’re poppin’ children out like tic tacs?

ERIC
Calm down, Carlos. He didn’t mean anything by it. Where in California are you from?

CARLOS

Pasadena.

MOON
Isn't that a pretty wealthy neighborhood?

CARLOS
I guess. I don't know; suppose we do okay.

CHAD
So why'd you come all the way to Nebraska for college? Are you here on like the Obama scholarship?

EVERYONE reacts with simultaneous responses.

SCENE 3: Gender

QIZHEN
You want to go next? (indicates ERIC)

ERIC
My name is Eric.

QIZHEN
So where are you from, Eric?

ERIC
I'm from Minneapolis…

QIZHEN
[interrupting]
Wait. I'm sorry; I didn't mean to cut you off. I just remembered I can't find your name on Canvas.

ERIC
Does Canvas say Eileen?

QIZHEN
Yes it does.

ERIC
My name's not Eileen, my name is Eric.

JESSIE
Why is that?

ERIC

It’s none of your business. I prefer that you use my name, which is Eric.

QIZHEN
I’m sorry. If I knew, I would have called you by the right name.

ERIC
I appreciate that.

CHAD
You look like an Eileen to me.

QIZHEN
It’s 2020, we should be able to use our own names on all our platforms...

ERIC;
...and my ID, and my drivers license.

EVERYONE reacts with simultaneous responses.

SCENE 4: International Student/Foreign Culture

JESSIE
Ok I think I’ll go next. I’m Jessie.

ERIC
Jessie? Aren’t you Indian?

JESSIE
Yes, I am.

CARLOS
Then how come you don’t have an Indian name?

JESSIE
I am Indian, and my name is Jessie because I’m a Christian

CHAD
Christian huh? I thought all Indians were Hindi

JESSIE
(loud sigh) FYI-Hindi is the official language. Hindu is the religion. But we embrace all religions in India.

QIZHEN
Your English sounds different. Like I have heard it somewhere. Did you work in a call center before?

JESSIE

Well, no. But can I help you with something?

EVERYONE reacts with simultaneous responses.

SCENE 5: Disability

MOON

Hi, my name is Moon. I’m from Norfolk. I just wanted to let everyone know that I have a hearing issue. I may ask you to repeat yourself if I have trouble hearing you.

CARLOS

So like do you sign or use Braille? You don’t sound like the people on TV who are deaf. They always sound funny.

MOON

No, I don’t sign. Braille is for people who can’t see to help them read. My hearing loss is only in my left ear so that may be why I don’t have a speech impediment.

CARLOS
You should wear a hearing aid so you don’t have an issue or that surgery thing…”cocky” something.

MOON
I think you mean a Cochlear implant and neither one of those is a viable option for me. It’s okay to just be deaf or hard of hearing. I’ve learned to adapt as I was born this way.

ERIC
What’s it like to be deaf?

MOON
Well I’m not totally deaf. I don’t know what it’s like to not be hard of hearing. It’s the same as being born with brown eyes. It just is your reality.

CHAD
(raising voice like he/she thinks the person didn’t hear)
But like how do you listen to music? Or are there sounds you can’t hear? Come on you have to be able to tell us what it’s like.

EVERYONE reacts with simultaneous responses.

SCENE 6: Nebraska Culture

CHAD
My name is Chad, I’m from Crawford.

CARLOS
I’ve never heard of Crawford, where is that?

CHAD
Way up in the northwest corner of the state.
[phone buzzing, dudebro looks at phone]

CHAD
Sorry, it’s my girlfriend. She is still there and I really miss her a lot.

CARLOS
When’s the next family reunion? Is it hard to find a girlfriend with all the brother-cousins around?

CHAD
Very funny.

JESSIE

So uh..what do people in Crawford do for fun?

CHAD
I played baseball all through grade school and high school

ERIC
I played softball in high school. I’d hate to lose balls in the cornfield.

CHAD
Well I grew up on a farm, but we don’t all wear overalls and have missing teeth, and the baseball field was actually right next to the golf course.

QIZHEN
Between the ol’ swimmin’ hole and the golf course.

EVERYONE reacts with simultaneous responses.
